# Informant-based screening tools for diagnosis of dementia, an overview of systematic reviews of test accuracy studies protocol

**DOI:** 10.1186/s13643-020-01530-3

**Published:** 2020-11-26

**Authors:** Martin Taylor-Rowan, Sara Nafisi, Amit Patel, Jennifer K. Burton, Terry J. Quinn

**Affiliations:** 1 Institute of Cardiovascular and Medical Sciences, University of Glasgow, Glasgow, Scotland; 2 Department of Health Science, University of Leicester, Leicester, England

**Keywords:** Dementia, Cognitive impairment, Diagnostic accuracy, Informant, Screening, Systematic review, Protocol, Review, Overview

## Abstract

**Background:**

Robust diagnosis of dementia requires an understanding of the accuracy of the available diagnostic tests. Informant questionnaires are frequently used to assess for dementia in clinical practice. Recent systematic reviews have sought to establish the diagnostic test accuracy of various dementia informant screening tools. However, most reviews to date have focused on a single diagnostic tool and this does not address which tool is ‘best’. A key aim of the overview of systematic reviews is to present a disparate evidence base in a single, easy to access platform.

**Methods:**

We will conduct an overview of systematic reviews in which we ‘review the systematic reviews’ of diagnostic test accuracy studies evaluating informant questionnaires for dementia. As an overview of systematic reviews of test accuracy is a relatively novel approach, we will use this review to explore methods for visual representation of complex data, for highlighting evidence gaps and for indirect comparative analyses. We will create a list of informant tools by consulting with dementia experts. We will search 6 databases (EMBASE (OVID); Health and Psychosocial Instruments (OVID); Medline (OVID); CINAHL (EBSCO); PSYCHinfo (EBSCO) and the PROSPERO registry of review protocols) to identify systematic reviews that describe the diagnostic test accuracy of informant questionnaires for dementia. We will assess review quality using the AMSTAR-2 (Assessment of Multiple Systematic Reviews) and assess reporting quality using PRISMA-DTA (Preferred Reporting Items for Systematic Review and Meta-analysis of Diagnostic Test Accuracy Studies) checklists. We will collate the identified reviews to create an ‘evidence map’ that highlights where evidence does and does not exist in relation to informant questionnaires. We will pool sensitivity and specificity data via meta-analysis to generate a diagnostic test accuracy summary statistic for each informant questionnaire. If data allow, we will perform a statistical comparison of the diagnostic test accuracy of each informant questionnaire using a network approach.

**Discussion:**

Our overview of systematic reviews will provide a concise summary of the diagnostic test accuracy of informant tools and highlight areas where evidence is currently lacking in this regard. It will also apply network meta-analysis techniques to a new area.

**Supplementary Information:**

The online version contains supplementary material available at 10.1186/s13643-020-01530-3.

## Background

Dementia is a prevalent and ever-increasing public health issue [1–3]. Depending upon the case definition applied, global prevalence of dementia is estimated to be 50 million [4].

The key to the effective management of dementia is early diagnosis. The ideal method for diagnosing dementia involves comprehensive multidisciplinary assessment informed by supplementary information such as neuropsychological testing, neuroimaging or tissue biomarkers [5]. However, this time-consuming approach requires specialist hospital-based services that are a limited resource in high-income countries. Thus, the ‘ideal’ approach to assessment is only feasible for a small proportion of the potential population affected.

In practice, a two-stage process is typically employed, with an initial screening process—often carried out by non-specialists—that is used to identify those that require a more detailed assessment from a specialist [6]. Various tools are available to screen for cognitive impairment; the most common of which directly assess a person’s cognition via questions and/or ‘pencil and paper’ tasks [7]. Such a process provides a ‘snapshot’ of cognitive function and does not capture cognitive decline; yet, this is a key component of the dementia diagnosis. As dementia progresses, insight is often lost and it can be challenging to obtain evidence of cognitive changes from the patient themselves. An attractive approach is to use informants (e.g. formal carers or relatives) with sufficient knowledge of the patient as a means of identifying any temporal change in cognition and related function.

There are a number of informant-based interview tools available that are designed to identify cognitive decline over time. Recent systematic reviews of the psychometric properties of commonly employed tools have assessed the Informant Questionnaire on Cognitive Decline in the Elderly (IQCODE) [8], the 8-item interview to Ascertain Dementia (AD8) [9] and the General Practitioner Assessment of Cognition (GPCOG) [10]. The growing number of dementia screening reviews published across various sources can be difficult for stakeholders to keep up with.

This overview of systematic reviews will draw together information regarding the diagnostic properties of all reviewed informant-based interview tools. Our primary intention is to create a summary of the existing evidence that could help clinicians in selecting an assessment that could help researchers in planning future test accuracy studies and that could help policy makers in forming recommendations around cognitive screening.

### Condition of interest and reference standard

The target condition for this diagnostic test accuracy overview of systematic reviews is all-cause dementia (clinical diagnosis). Dementia is a syndrome characterised by progressive cognitive or neuropsychological decline that is sufficient to interfere with usual functioning. The clinical diagnosis of dementia is established through a personal history from the person and/or suitable collateral sources in combination with direct examination, via cognitive assessment and supplementary information from laboratory and radiology investigations.

The ideal test pathway would involve a person self-presenting or being referred to a dedicated memory service for multidisciplinary assessment that would often involve clinical examination, repeated cognitive testing over time and laboratory and imaging testing. While the approach to diagnosis may vary across differing healthcare systems, comprehensive assessment and demonstrating functionally important change in cognition over time is essential. Relatives, friends and caregivers are well placed to comment on change over time and functional consequences. The diagnostic assessment would, where possible, include collateral information often in the form of a detailed interview. The informant screening tools attempt to capture the most discriminating aspects of this interview in a short, standardised questionnaire.

There are various internationally accepted dementia diagnostic criteria that help operationalise and standardise the assessment. The most common of which are the various iterations of the World Health Organization International Classification of Diseases (ICD) ICD-10 [11] and the American Psychiatric Association Diagnostic and Statistical Manual of Mental Disorders (DSM) for all-cause dementia and subtypes [12]. The label of dementia encompasses varying pathologies, and specific diagnostic criteria are also available for each specific dementia subtype, e.g. NINCDS-ADRDA (National Institute of Neurological and Communicative Disorders and Stroke and the Alzheimer’s Disease and Related Disorders Association) criteria for Alzheimer’s dementia [13, 14]; McKeith criteria for Lewy Body dementia [15]; Lund criteria for frontotemporal dementias [16]; and the NINDS-AIREN (National Institute of Neurological Disorders and Stroke Internationale pour la Recherche et l’Enseignement en Neurosciences) criteria for vascular dementia [17].

The syndrome of mild cognitive impairment (MCI) may also be considered in studies looking at cognitive screening. Varying definitions of MCI have been proposed, usually based on the demonstration of cognitive deficits beyond what would be expected for age but with no evidence of functional impairment from these deficits [18]. MCI is often conceptualised as a precursor to overt dementia, but progression to dementia from MCI is neither inevitable nor predictable [19]. While our primary focus will be dementia, we will also consider studies looking at test properties of informant tools against an MCI diagnosis. The suitability of informant questionnaires for the assessment of MCI is uncertain. As the method adopted by informant questionnaires to establish cognitive impairment is predominantly via reports of everyday functional impairment, this might suggest that they are incompatible with the MCI concept; however, despite this, a number of studies have sought to evaluate the value of informant tools to improve MCI diagnosis in memory clinic settings [20, 21]. Better establishing the evidence base for use of informant tools to detect MCI would therefore help to clarify if the use of informant tools for assessment of non-dementia level cognitive impairment is appropriate.

Our focus is on informant-based cognitive screening tests. In practice, these tests would not be used in isolation to a make a clinical diagnosis of dementia. It could be argued that the target condition being ‘diagnosed’ by the screening test is a state of possible abnormal cognition that requires more detailed assessment. To date, the dementia test accuracy literature has been based on a more traditional paradigm of comparing the index test to the final diagnosis of dementia/no dementia [22] and so we have retained this approach for our review.

### Index test

Our index test will be any informant-based interview tool intended to identify cognitive decline. These typically consist of questionnaires that are directed to a spouse, family member or carer. The most commonly used informant assessments are the IQCODE and the AD8. Some tests combine informant interviews with direct to patient assessment, for example the GPCOG.

There are many reasons to favour the use of informant assessments in dementia screening. Such tools typically have an immediacy and relevance, which appeals to its users. In addition, the assessment and scoring process is usually brief (e.g. < 10 mins) and requires minimal training. Moreover, there are data to suggest that, by comparison to standard direct cognitive assessments, informant interviews may be less influenced by cultural and educational biases [23]. Diagnostic criteria for dementia make explicit reference to documenting decline as well as the involvement of collateral informants, emphasising the potential utility of an informant interview tool.

Our index tests are screening tools, rather than diagnostic instruments. However, for consistency with other test accuracy reviews we have retained the descriptor ‘diagnostic test accuracy’ for describing the methods used in our overview of systematic reviews.

### Why an overview of systematic reviews?

A thorough understanding of the diagnostic properties of informant tools would enable a more informed approach to testing. Critically evaluating the current evidence-base regarding the accuracy of informant-based screening tools is essential to this process. A number of reviews of the diagnostic properties of informant-based tools have been produced [24–27]; however, this rapidly growing body can be overwhelming for clinicians and decision-makers to fully absorb. Furthermore, these reviews typically focus upon the properties of informant-based screening tools in isolation; hence, it is currently difficult to compare the properties of respective tools and form valid conclusions as to the suitability of one tool over another, for instance in a particular setting. Arguably, a synthesis of reviews is therefore needed to present a broader picture of the evidence base, which can then be used to inform the utilisation of these tools in clinical practice. We will also use this overview of systematic reviews to ‘map’ the existing evidence and highlight gaps in the literature for given tools and specific settings.

The motivation for producing an overview of systematic reviews at this time is twofold. Priority setting work with people living with dementia has emphasised the importance ascribed to the diagnostic approach. To ensure that diagnosis is as accurate as possible, we felt it would be useful to collate and compare all the relevant reviews on a particular approach to assessment [28]. Secondly, the Cochrane Dementia and Cognitive Improvement Group have produced a suite of reviews on informant tests and so we know there is contemporary literature on which to base an overview of systematic reviews. Individual reviews of test accuracy have been used in recent national guidelines [5]; but a comment from an end-user was that having all the evidence in one collection would be preferable.

## Methods

### Aims and objectives

Our over-arching aim is to produce an overview of systematic reviews that offers a synthesis of all systematic reviews of informant-based cognitive screening tools. Using the terminology of test accuracy research, our primary question is: *what is the diagnostic accuracy of the various informant based cognitive screening tools for making a diagnosis of dementia across various settings and populations?*

### Secondary objectives

If data allow, we hope to use the overview of systematic reviews to inform a number of secondary objectives:

- To map the availability and quality of evidence regarding accuracy of informant-based tests.
- To identify gaps in the evidence base where reviews or primary research are needed.
- To summarise accuracy across specific settings (secondary care, primary care, community) and populations (neurological disease, delirium, MCI).
- To compare informant tests with the intention of drawing inferences about comparative accuracy.

### Design

We used the PRISMA-P (Preferred Reporting Items for Systematic Review and Meta-Analysis Protocols) checklist for reporting in this overview of systematic reviews protocol.

We appreciate that the design, conduct and interpretation of an overview of systematic reviews is evolving [29]. Our approach to the overview of systematic reviews was informed by the Cochrane Handbook [30] and also by recent methodological reviews of the overview of systematic reviews process [31], discussions with authors of previous and ongoing overviews of systematic reviews [32] and a UK meeting held by the National Institute of Health Research Complex Reviews Support Unit (NIHR CRSU) with a focus on complex review methods. An initial draft of this protocol was considered by the Cochrane Test Accuracy Methods Group. The group offered useful comments and suggestions that have been incorporated into the protocol.

### Inclusion and exclusion criteria

We will include any systematic reviews of the literature meeting our selection criteria. There are no standard, consensus criteria that define a review as ‘systematic’ for the purposes of an overview of systematic reviews. For our overview of systematic reviews, to distinguish a systematic review (eligible) from a non-systematic literature review (ineligible), the review must contain a description of a literature search that used more than one electronic database, must give explicit inclusion and exclusion criteria, and must offer some attempt at critical appraisal of the included studies.

Some overviews of systematic reviews have limited their scope to a specific evidence source, for example Cochrane reviews. As an intended outcome of our overview of systematic reviews is to produce an evidence ‘map’, we considered it important to include all relevant reviews. We hope that this inclusive approach will allow for as comprehensive a picture of the current literature as possible.

In designing an overview of systematic reviews, a priori decisions need to be made around whether the authors will update eligible reviews with relevant research published after the review. Our overview of systematic reviews will only include the published systematic review data. We will not update reviews with additional data from contemporary studies, and where there is no existing review, we will not create one for the purposes of this overview of systematic reviews. We felt this was appropriate as there are a number of recently published reviews in our topic area and the evidence base around test accuracy is more ‘stable’ over time than the evidence base for a novel intervention. We will also avoid correcting imperfections identified in reviews (i.e. lack of risk of bias assessment for included studies) but rather will note when these are absent.

To be eligible for inclusion, reviews must meet the following criteria:

1. Performed a systematic search of the published literature for at least one informant-based cognitive screening tool.
2. Primarily interested in investigating the diagnostic properties (test accuracy) of an informant-based cognitive screening tool(s) and report both sensitivity and specificity statistics of at least one informant-based tool.

Thus, our overview of systematic reviews will not include reviews, editorial or opinion pieces, based on only a selection of possible literature. There are various approaches to presenting review data. To be included, the review should offer, at least, a narrative synthesis with a focus on the informant test accuracy and/or a quantitative summary of test accuracy data and/or give a structured assessment of the ‘quality’ of the included studies.

We will make no exclusions on the basis of methodological quality, use of best practice methods or approach to data synthesis. Data may allow us to explore these factors and compare them at the review level. Where our search highlights a potentially relevant review that is only available as an abstract, we will contact the authors to verify whether a full paper is available.

### Describing test setting and populations of special interest

Screening for dementia takes place in various settings and at different stages in the dementia pathway. In this overview of systematic reviews, we will only consider reviews of informant tools that were utilised as part of an initial assessment for cognitive decline; however, we will not restrict by setting. We will operationalise the settings in which informant tools are used as primary care, secondary care and community.

Primary care shall be defined as settings in which the patient presents to a non-specialist service, such as general practice, because of subjective memory complaints or because of concerns from others. Patients seen in primary care are unlikely to have had any previous cognitive assessment. Employment of informant-based interview tools in these cases can be referred to as ‘triage’ or ‘case-finding’.

Secondary care shall be defined as any settings where patients are referred for expert input and includes general hospitals and more specialist settings such as memory clinics. Screening in such settings typically involves opportunistic screening of adults presenting as unscheduled admissions, or, in the case of more specialist settings, part of a wider pre-planned assessment, designed to distinguish those who have dementia from those who do not. Case-mix in these settings will include patients with comorbid physical and psychiatric diagnoses, and prevalence rates of dementia are typically high. Patients admitted to secondary care are more likely to have had cognitive assessment prior to referral; albeit this is not always the case [33]. They may also have had other assessments such as neuroimaging or laboratory tests.

Within the secondary care rubric, we have identified particular populations of interest, where the informant based approach may have utility or may have differing test properties. People living with neurological disorders such as stroke, multiple sclerosis or Parkinson’s disease have a high prevalence of cognitive problems but physical impairments may preclude direct to patient assessment with pencil and paper tests [34]. Patients with the clinical syndrome of delirium often have pre-existing cognitive impairment but a direct to patient test will be confounded by the delirium [35]. In these situations, informant assessment may be particularly useful.

Community settings shall be defined as settings in which the cohort is unselected, i.e. ‘population screening’. Prevalence of dementia is likely to be lower in such settings than that which is found in secondary or primary care.

### Process to identify relevant index tests

We identified tools of interest for our overview of systematic reviews via a multi-stage process: a group consultation with experts who had extensive experience in the use of informant tools for assessing dementia/cognitive impairment, supplemented by scoping the literature online.

### Search methods for identification of reviews

We propose a search strategy with three complementary approaches. We hope this will ensure that our overview of systematic reviews is suitably comprehensive.

A) We will perform title searching of all reviews and protocols in the Cochrane Dementia and Cognitive Improvement portfolio. We will contact the authors of any relevant protocols where a complete review is not published.
B) As part of their ongoing dementia diagnostic test accuracy work, the Cochrane Dementia and Cognitive Improvement Group run a sensitive search across a variety of literature databases. This regularly updated search is designed to capture all diagnostic test accuracy studies with a focus on neuropsychological assessment. We will work with the latest of these searches looking for any systematic review titles [36].
C) Using the methods outlined in the earlier section, we have identified a list of relevant informant assessments. The assessments of interest are AD8, Blessed Dementia Scale [37], GPCOG, IQCODE, Deterioration Cognitive Observee [38], Dementia Questionnaire [39], Short Memory Questionnaire [40], Symptoms of Dementia Screener [41] and Concord Informant Dementia [42]. We will perform a systematic search for these tests (see Supplementary materials). The search will use the test name and any synonyms and combine with a validated search filter for systematic reviews. The process will be iterative: if, during the course of the review, we find an informant-based test that was not included in our original list, then we will add this to the list and perform a further systematic search. This third search will include the following databases: EMBASE (OVID), Health and Psychosocial Instruments (OVID), Medline (OVID), CINAHL (EBSCO), PSYCHinfo (EBSCO) and the PROSPERO registry of review protocols. All databases will be searched from inception to present.

We will contact authors working in the field of dementia test accuracy to identify other relevant systematic reviews. Should an identified review be in a non-English language, we will arrange translation. We will contact authors of grey literature that is identified by our search and access theses from institutions known to be involved in prospective dementia studies. However, we will only include reviews that have been published in peer reviewed scientific journals in our final review. We will study reference lists of all identified reviews in order to identify additional titles not found by our search and will repeat this process until no new titles are found [43].

## Data collection and analysis

### Selection of reviews

Search results will be imported into Covidence software [44] for deduplication and screening. Two authors (MT and SN) will independently assess titles and abstracts identified by our searches and exclude obviously irrelevant reviews. The same two authors will then read full texts of remaining titles and select reviews that meet our inclusion criteria. In the event of disagreement between authors, a third, neutral author (JB) shall act as an arbiter if consensus is not reached through discussion. This process will be illustrated in a PRISMA flow diagram.

### Data extraction and management

Two authors (MT and SN) will extract data independently. Disagreements will be resolved by discussion, with assistance from a third author (TQ), if necessary. Data will be extracted on to a data collection proforma that has been specifically designed by the author team and piloted on two exemplar reviews—one from Cochrane [26] and one from non-Cochrane [45]. Specifically, we will extract and record the details of date of last literature search; the aims and rationale; primary tools searched for; included studies; population of interest; setting (including countries studied in primary papers); tools evaluated (along with sensitivity and specificity values of said tools and thresholds evaluated if appropriate); total number included in the review and total number with dementia; subgroups within the study; and methods used for assessing ‘quality’ of included studies (see Supplementary materials).

We anticipate that reviews will use differing approaches to present their data. Where meta-analytical data are presented, we will extract the summary measures but will also record the individual test level data reported in each review as this will be used to compare tests via a network meta-analysis. If only a narrative summary is offered, we will extract the authors’ conclusions on test accuracy. We also recognise that approaches to the critical appraisal of included studies will differ between reviews. We will present a summary of the ‘quality’ of individual studies included in the review using the approach employed in the primary review.

### Assessment of methodological quality of included reviews

Two authors (MT and SN) will independently assess the methodological quality of included reviews, basing this assessment on the AMSTAR-2 (assessment of multiple systematic reviews) measurement tool [46] and considering the following key domains.

- Clarity of review objective.
- Description of study eligibility criteria.
- Extent of searching undertaken.
- Transparency of assessment process.
- Assessment of publication bias.
- Assessment of heterogeneity.

The AMSTAR-2 tool offers a series of statements to guide the assessment. These have been framed in terms of intervention reviews. Where relevant, we have modified this guidance to make the materials appropriate for assessment of test accuracy research. For example, where the original tool assesses use of the Patients, Interventions, Control, Outcomes (PICO) categorisation, our revised tool looks for evidence of index test, reference standard and setting categorisation (see Supplementary materials).

We will pilot our revised AMSTAR-2 against two reviews (one Cochrane [26] and one non-Cochrane [45]) to see if any further study specific modifications are needed.

We recognise that in primary diagnostic test accuracy research, assessment of methodological quality is often complicated by poor reporting. The same may be true of diagnostic test accuracy reviews. We will complement our AMSTAR-2 assessment with a specific assessment of the reporting of included reviews. The same two reviewers will evaluate the reporting standard of each review by utilising the PRISMA-DTA (Preferred Reporting Items for a Systematic Review and Meta-analysis of Diagnostic Test Accuracy Studies) checklist [47].

When authors on this overview of systematic reviews are also authors of an included review, they will not be involved in assessment of methodological or reporting quality of that review. This will be done independently by two other authors with access to an independent arbitrator as needed.

### Data synthesis

Primary outputs from our overview of systematic reviews will be tables of included reviews for each informant assessment tool and an evidence ‘map’ describing the systematic review data available for each tool in each setting and population of interest. The map will take a tabulated form, with columns representing each informant assessment tool and rows representing the settings and special populations of interest (secondary care, primary care, community, neurological disease, delirium and MCI). Within each resulting cell, we will describe the systematic review(s) available, the summary measure of accuracy (if available), the risk of bias of included studies and the AMSTAR-2 rating for the review. While we have a plan for illustrating the data, we appreciate that the data may not be suited to the approach outlined here. We will explore other methods of visual presentation of our acquired data.

If data are suitable, we propose quantitative analysis of test accuracy using MetaDTA version 1 [48]. We will calculate, where possible, summary estimates of test accuracy for each ‘cell’ of the evidence map. We are principally interested in the test accuracy of the informant tools for the dichotomous variable ‘dementia/no dementia’. We will present summary sensitivity, specificity, predictive values and/or and likelihood ratios. These data will be combined using the bivariate method [49].

We recognise that the statistical techniques available for creating aggregate test accuracy data have evolved since the first test accuracy reviews were published. If the included review already presents a quantitative analysis, we will produce our own summary estimates using the individual study level data contained within the review. If a ‘cell’ has two reviews that contain differing studies, we will include all relevant studies in our summary estimate. The quantitative test accuracy summary analyses will be repeated and restricted to studies at low risk of bias (based on individual study level data within the included review).

As a final step, if the data allow, we will attempt indirect comparative analyses using a network-based approach and ranking the tools based on likely superiority in terms of sensitivity and specificity [50].

We will then employ a GRADE (Grading of Recommendations Assessment, Development, and Evaluation) [51] approach to establish overall strength of evidence for the use of informant tools to screen for dementia, following recommended guidelines for applying GRADE to diagnostic tests [52].

## Discussion

We anticipate seven potential issues in the selection and collation of systematic reviews into our overview of systematic reviews. We outline approaches to these issues that have been developed in discussion with authors of existing overview of systematic reviews and with systematic review methodologists.

Overlapping reviews: We will not exclude any review that focuses on the same test as a review already included. Similarly, we will not exclude any review that includes studies that also feature in another included review. If we identify overlapping reviews, we will quantify the extent of overlap (noting the reference of each study included in both reviews). If we find reviews with the same test and setting/population, we will compare (where available) the review results, describing differences in summary accuracy, ‘quality’ assessment, and conclusions of the review authors. Depending on the nature of the review data, this comparative exercise may be presented as a table or as narrative text.

Discrepant results: Related to the issue of overlapping reviews is the situation where two reviews ask the same question but present seemingly discrepant results. We will explore potential reasons for this by comparing search strategies, inclusion/exclusion criteria, critical appraisal methods used within the review and method used for evidence synthesis.

Forms of bias in individual studies: Informant tool performance may vary based upon particular types of bias, such as the type of person doing the test or the time lag duration between index test and reference standard. We will not explore these possibilities individually in our overview of systematic reviews, but rather will rely upon the risk of bias assessments of included reviews. If reviews do not conduct risk of bias assessments that sufficiently consider such forms of bias, we will highlight this issue in our overview of systematic reviews.

Historical reviews: Diagnostic test accuracy of an informant questionnaire is unlikely to become outdated or superseded by a new method. Thus, unlike overviews of systematic reviews of interventions where historical reviews may be misleading, we will operate no restrictions based on the ‘age’ of the review. We will note the dates of the search. Where a review has been superseded by an update review, we will include the most contemporary review.

Author’s own reviews: We anticipate that many of the included reviews will be part of the suite of diagnostic test accuracy reviews performed by the Cochrane Dementia and Cognitive Improvement Group. Some of these reviews will have been authored by authors of this overview of systematic reviews. We will attempt to ensure objectivity and avoid any potential conflicts by pre-specifying methods in this protocol, by using validated and operationalised assessment tools and by making sure that the researchers appraising and extracting data were not part of the author team on the included review.

Part relevant reviews: There may be systematic reviews on a broader topic, for example cognitive screening tests in general, which include informant-based tests. We will consider reviews where informant assessments are included but not the focus of the review if they otherwise are aligned with inclusion and exclusion criteria. As described in our general inclusion/exclusion criteria, for the review to be eligible it must offer a synthesis of data specific to the informant tool(s) included.

Reviews that cover multiple settings/domains: When constructing our evidence map, we may encounter reviews that describe evidence over multiple settings, conditions, or populations, and thus do not easily fit into one particular category. In this circumstance, we will assign a review into each category that is described in that review. While this may indicate that more reviews are available than in reality, we will try to mitigate this perception by providing a reference list for each cell so as to highlight when a single review has been placed in multiple categories. We will also explore alternate methods of constructing our evidence map, based upon the evidence we acquire.

We hope that this overview of systematic reviews will be of interest to differing groups. Previous overviews of systematic reviews of diagnostic test accuracy for dementia exist [53], but they focus solely on the methodological quality of available reviews and do not provide a concise summary of reported test accuracy. These older diagnostic test accuracy overviews of systematic reviews describe the quality of systematic reviews as being suboptimal. Our overview of systematic reviews will provide an update that will determine if the standard is improving.

In addition, the clinical content described in our overview of systematic reviews should be useful to clinicians and policy makers as it allows a synthesis of the accuracy of various test approaches. The evidence map, in particular the evidence gaps, will be of interest to researchers and funders as it may highlight areas to prioritise future test accuracy research. The process and approaches used to create the overview of systematic reviews will be of interest to the evidence synthesis community. Overview of systematic reviews of test accuracy is a novel area, with no consensus on the best approach. This overview of systematic reviews will serve as an example of a particular method that could be applied to other topic areas.

## Supplementary Information

**Additional file 1.** Supplementary materials. Search terms.

## Data Availability

Data that inform the review will all be presented within the primary publication.

## Abbreviations

USA: United States of America
NICE: National Institute for Health and Care Excellence
IQCODE: Informant Questionnaire on Cognitive Decline in the Elderly
AD8: Ascertain Dementia 8
GPCOG: General Practitioner Cognitive Screen
ICD: International Classification of Diseases
DSM: Diagnostic and Statistical Manual of Mental Disorders
NINCDS-ADRDA: National Institute of Neurological and Communicative Disorders and Stroke and the Alzheimer’s Disease and Related Disorders Association
MCI: Mild cognitive impairment
PRISMA-P: Preferred Reporting Items for Systematic Review and Meta-Analysis Protocols
NIHR CRSU: National Institute of Health Research Complex Reviews Support Unit
AMSTAR-2: Assessment of Multiple Systematic Reviews
PICO: Patients, Interventions, Control, Outcomes
PRISMA-DTA: Preferred Reporting Items for Systematic Review and Meta-analysis of Diagnostic Test Accuracy Studies
GRADE: Grading of Recommendations Assessment, Development, and Evaluation
