# Supplementary material for: Informant-based screening tools for diagnosis of dementia, an overview of systematic reviews of test accuracy studies protocol
Source: Syst Rev. 2020 Nov 26;9:271. doi: 10.1186/s13643-020-01530-3 (PMC7694897; doi:10.1186/s13643-020-01530-3)
Supplement: Supplementary file 1 — Additional file 1. Supplementary materials. Search terms. [file 13643_2020_1530_MOESM1_ESM.docx]

**Supplementary materials**

**Search terms**

1. IQCODE.mp.

2. AD8.mp.

3. AD-8.mp.

4. GPCOG.mp.

5. SED.mp.

6. AQ.mp.

7. "Observation List of Possible Early Signs of Dementia".mp.

8. "Informant Questionnaire for Cognitive Decline in the Elderly".mp.

9. "Ascertain Dementia 8 item questionnaire".mp.

10. "General Practice Cognitive Screener".mp.

11. "Symptoms of Early Dementia Questionnaire".mp.

12. "Alzheimer’s Questionnaire".mp.

13. “Deterioration cognition observe”.mp.

14. “Blessed dementia scale”.mp.

15. “Concord informant dementia scale”.mp.

16. “Short memory questionnaire”.mp.

17. “Symptoms of dementia screener”.mp.

18. “Brief cognitive rating scale”.mp.

19. “Dementia questionnaire”.mp.

20. 1 or 2 or 3 or 4 or 5 or 6 or 7 or 8 or 9 or 10 or 11 or 12 or 13 or 14 or 15 or 16 or 17 or 18 or 19

21. MEDLINE.tw.

22. exp systematic review/ or systematic review.tw.

23. meta-analysis/

24. intervention$.ti.

25. 21 or 22 or 23 or 24

26. 20 and 25

**Proforma**

| **Review** | **Primary tools searched for** | **Primary setting focus** | **Sensitivity** | **Specificity** | **Included studies** | **Total number included in review** | **Total number with dementia** | **Method used to assess study quality** | **Subgroups within review** | **Population of interest** | **Date of last search** | **Aims and rational & Review Summary of evidence** |
| --- | --- | --- | --- | --- | --- | --- | --- | --- | --- | --- | --- | --- |
|  |  |  |  |  |  |  |  |  |  |  |  |  |
|  |  |  |  |  |  |  |  |  |  |  |  |  |
|  |  |  |  |  |  |  |  |  |  |  |  |  |
|  |  |  |  |  |  |  |  |  |  |  |  |  |
|  |  |  |  |  |  |  |  |  |  |  |  |  |
|  |  |  |  |  |  |  |  |  |  |  |  |  |
|  |  |  |  |  |  |  |  |  |  |  |  |  |

**AMSTAR-2**

**PRISMA-P checklist**

This checklist has been adapted for use with protocol submissions to *Systematic Reviews* from Table 3 in Moher D et al**:** Preferred reporting items for systematic review and meta-analysis protocols (PRISMA-P) 2015 statement. *Systematic Reviews* 2015 **4**:1

| **Section/topic** | **#** | **Checklist item** | **Information reported** | | **Line number(s)** |
| --- | --- | --- | --- | --- | --- |
|  |  |  | **Yes** | **No** |  |
| **ADMINISTRATIVE INFORMATION** | | | | | |
| **Title** | | | | | |
| Identification | 1a | Identify the report as a protocol of a systematic review | x |  | 3 |
| Update | 1b | If the protocol is for an update of a previous systematic review, identify as such |  | x | NA |
| **Registration** | 2 | If registered, provide the name of the registry (e.g., PROSPERO) and registration number in the Abstract |  | x | NA |
| **Authors** | | | | | |
| Contact | 3a | Provide name, institutional affiliation, and e-mail address of all protocol authors; provide physical mailing address of corresponding author | x |  | 4-12 |
| Contributions | 3b | Describe contributions of protocol authors and identify the guarantor of the review | x |  | 454-456 |
| **Amendments** | 4 | If the protocol represents an amendment of a previously completed or published protocol, identify as such and list changes; otherwise, state plan for documenting important protocol amendments |  | x | NA |
| **Support** | | | | | |
| Sources | 5a | Indicate sources of financial or other support for the review | x |  | 452 |
| Sponsor | 5b | Provide name for the review funder and/or sponsor | x |  | 452 |
| Role of sponsor/funder | 5c | Describe roles of funder(s), sponsor(s), and/or institution(s), if any, in developing the protocol | x |  | 452-453 |
| **INTRODUCTION** | | | | | |
| **Rationale** | 6 | Describe the rationale for the review in the context of what is already known | x |  | 47-78 |
| **Objectives** | 7 | Provide an explicit statement of the question(s) the review will address with reference to participants, interventions, comparators, and outcomes (PICO) | x |  | 163-175 |
| **METHODS** | | | | | |
| **Eligibility criteria** | 8 | Specify the study characteristics (e.g., PICO, study design, setting, time frame) and report characteristics (e.g., years considered, language, publication status) to be used as criteria for eligibility for the review | x |  | 189-258 |
| **Information sources** | 9 | Describe all intended information sources (e.g., electronic databases, contact with study authors, trial registers, or other grey literature sources) with planned dates of coverage | x |  | 266-295 |
| **Search strategy** | 10 | Present draft of search strategy to be used for at least one electronic database, including planned limits, such that it could be repeated | x |  | Supplementary materials |
| ***STUDY RECORDS*** | | | | | |
| Data management | 11a | Describe the mechanism(s) that will be used to manage records and data throughout the review | x |  | 307-324 |
| Selection process | 11b | State the process that will be used for selecting studies (e.g., two independent reviewers) through each phase of the review (i.e., screening, eligibility, and inclusion in meta-analysis) | x |  | 307-354 |
| Data collection process | 11c | Describe planned method of extracting data from reports (e.g., piloting forms, done independently, in duplicate), any processes for obtaining and confirming data from investigators | x |  | 307-354 |
| **Data items** | 12 | List and define all variables for which data will be sought (e.g., PICO items, funding sources), any pre-planned data assumptions and simplifications | x |  | 311-317 |
| **Outcomes and prioritization** | 13 | List and define all outcomes for which data will be sought, including prioritization of main and additional outcomes, with rationale | x |  | 81-119 |
| **Risk of bias in individual studies** | 14 | Describe anticipated methods for assessing risk of bias of individual studies, including whether this will be done at the outcome or study level, or both; state how this information will be used in data synthesis | x |  | 327-354 |
| ***DATA*** | | | | | |
| **Synthesis** | 15a | Describe criteria under which study data will be quantitatively synthesized | x |  | 357-383 |
|  | 15b | If data are appropriate for quantitative synthesis, describe planned summary measures, methods of handling data, and methods of combining data from studies, including any planned exploration of consistency (e.g., *I* ^2^, Kendall’s tau) | x |  | 357-383 |
|  | 15c | Describe any proposed additional analyses (e.g., sensitivity or subgroup analyses, meta-regression) | x |  | 357-383 |
|  | 15d | If quantitative synthesis is not appropriate, describe the type of summary planned | x |  | 357-366 |
| **Meta-bias(es)** | 16 | Specify any planned assessment of meta-bias(es) (e.g., publication bias across studies, selective reporting within studies) | X |  | 388-396 |
| **Confidence in cumulative evidence** | 17 | Describe how the strength of the body of evidence will be assessed (e.g., GRADE) | X |  | 400-403 |
